# Standardizing Vulvovaginal candidiasis diagnosis in Uganda: A case for British Association for Sexual Health and HIV 2019 guidelines

**DOI:** 10.1016/j.ijregi.2025.100735

**Published:** 2025-08-21

**Authors:** Bwambale Jonani

**Affiliations:** 1 Department of Immunology and Molecular Biology, School of Biomedical Sciences, College of Health Sciences, Makerere University, Kampala, Uganda; 2 Laboratory Department, Sebbi Hospital, Wakiso, Uganda

**Keywords:** Diagnosis, Prevalence, Uganda, Vulvovaginal candidiasis

## Abstract

Vulvovaginal candidiasis (VVC) significantly affects women’s health in Uganda, with an annual economic burden of 280.77 billion Ugandan Shillings (US$75,696 annually), which is 11.22% of health spending. Current clinical practices predominantly rely on syndromic management or, where available, Sabouraud Dextrose Agar cultures, which cannot distinguish between colonization and active infection, leading to misdiagnosis and inappropriate antifungal use. The 2019 British Association for Sexual Health and HIV guidelines offer a structured diagnostic framework that integrates clinical assessment, microscopy, and targeted culture techniques. This review proposes implementing these guidelines within Uganda’s laboratory hub network to improve diagnostic accuracy and antifungal stewardship.

## Highlights

• Uganda’s lab hub system offers a framework to implement British Association for Sexual Health and HIV 2019 guidelines.
• Reliance on fungal culture causes misdiagnosis and antifungal overuse.
• Standardization of Vulvovaginal candidiasis diagnosis would enhance antifungal stewardship in Uganda.

## Background

Vulvovaginal candidiasis (VVC) significantly affects women’s health in Uganda, where diagnostic approaches vary widely across different healthcare facilities. The condition manifests in two forms: acute cases presenting as isolated episodes and recurrent cases, defined by four or more symptomatic episodes within a twelve-month period [1]. While the global prevalence in women of reproductive age remains at 10-15%, sub-Saharan Africa reports markedly higher rates (33%), particularly during pregnancy [2].

Recurrent vulvovaginal candidiasis is estimated to cost Uganda US$75.696 annually, which is 11.22% of the annual health spending [3]. A meta-analysis of seven Ugandan studies [[4], [5], [6], [7], [8], [9], [10]] revealed a concerning pooled VVC prevalence of 46% (95% CI: 35-57%) **(**S1 File**)**. The diagnostic criteria across these studies showed significant inconsistencies, with six out of seven relying solely on culture methods, which proved insufficient in distinguishing between colonization and active infection. This limitation has led to potential overdiagnosis and unnecessary antifungal use, underscoring the urgent need for standardized diagnostic approaches.

## Current diagnostic landscape

Uganda’s healthcare system operates through a hierarchical structure designed to serve a population of approximately 45 million. Village health teams serve as the initial points of contact within communities. The formal healthcare facility hierarchy begins with Health Center II (HC II) facilities at the parish level, serving populations of approximately 5,000 people and providing basic preventive and outpatient curative services. Health Centre III (HC III) facilities operate at the sub-county level, serving approximately 20,000 people and offering additional services, including basic laboratory diagnostics and maternity care. Health Center IV (HC IV) facilities function at the county level, serving populations of approximately 100,000 people, and providing emergency surgery and comprehensive laboratory services. District and regional referral hospitals form the upper tiers, offering specialized services, and serve as referral centers for complex cases.

Within this structure, the Ministry of Health has established an innovative network of 100 laboratory hubs strategically positioned to serve facilities within a 40-km radius [11]. This hub system aims to bridge the diagnostic capacity gap between different facility levels (S2 Figure). Although this established infrastructure has not yet been specifically utilized for VVC diagnosis, it presents a promising opportunity to implement standardized diagnostic approaches. Lower-level facilities often rely solely on clinical assessment, while higher-level facilities predominantly use Sabouraud Dextrose Agar culture as their primary diagnostic tool, occasionally supplemented by microscopy where available.

However, this diagnostic approach has several limitations in clinical settings. The inability to differentiate between colonization and active infection poses a fundamental challenge, often leading to misdiagnosis. Furthermore, species-level identification remains limited, quality control measures vary considerably across facilities, and access to diagnostic resources differs markedly between urban and rural settings. In rural areas, limited laboratory infrastructure and expertise create noticeable disparities in diagnostic capabilities, compromising the accuracy and timeliness of VVC diagnosis. In Uganda, the 2023 Clinical Guidelines recommend topical therapy as the first-line treatment for vulvovaginal candidiasis (clotrimazole pessaries 100 mg nightly for 6 days or nystatin pessaries 100,000 IU nightly for 10 days), reserving fluconazole (150–200 mg daily for 5 days) for recurrent cases [12]. In practice, however, fluconazole is widely available in pharmacies and private drug shops, often without a prescription. Pharmaceutical sector assessments indicate that Uganda’s healthcare delivery system is heavily dependent on private sector medicine outlets, with approximately 62% of antifungal treatments for VVC obtained from private vendors [13]. The most common formulation is the 150 mg oral capsule, marketed for single-dose treatment, although 50 mg and 200 mg tablets and oral suspensions are also available. A single 150 mg capsule typically costs less than 3000 UGX (≈0.8 US$), making it highly affordable and accessible. This widespread over-the-counter access has encouraged self-medication and empiric dispensing without laboratory confirmation, diverging from national guideline recommendations and contributing to inappropriate use and the emergence of fluconazole resistance in vaginal *Candida* isolates.

## British Association for Sexual Health and HIV 2019 guidelines: a framework for improvement

### Microscopy requirements

These guidelines establish microscopy as a crucial component of the initial diagnosis of VVC. Healthcare providers are required to obtain a high vaginal swab (HVS) of vaginal discharge for Gram staining and phase-contrast wet-film microscopy. This examination should identify specific fungal elements, such as blastospores, pseudohyphae, and neutrophils, which indicate an ongoing *Candida* infection. The presence of only blastospores and neutrophils suggests infection by non-albicans species. The presence of neutrophils in vaginal secretions signifies an inflammatory response, indicating an active infection, whereas the presence of *Candida* without neutrophils likely indicates colonization.

## Culture methods

The guidelines recommend against the use of fungal culture as the primary diagnostic tool for acute VVC because of its lack of cost-effectiveness and its reliability in differentiating between colonization and active infection. In addition, some women with symptomatic VVC may test culture-negative, often due to prior antifungal exposure or limitations in culture sensitivity, further underscoring the need for combined clinical and microscopic assessment. Clinical evaluation and microscopy should be the focus of diagnosing acute VVC. However, fungal culture remains an important tool for diagnosing recurrent VVC. In such cases, it is recommended to use HVS and directly plate the samples onto solid fungal growth media, such as Sabouraud Dextrose Agar, for accurate growth quantification and result interpretation. If direct plating is not possible owing to a lack of fungal culture facilities, HVS samples can be placed in a suitable transport medium, although quantification may become unreliable after 12 hours because of continued fungal growth.

### Implementation framework for Uganda

The proposed implementation strategy leverages Uganda’s existing laboratory hub network through a carefully structured approach across healthcare levels. At the parish level, HC II facilities would focus on standardized symptom screening and basic clinical assessment, with established pathways for referring complex cases to higher-level facilities (Table 1).

**Table 1:** Recommended diagnostic and clinical management of vulvovaginal candidiasis at different healthcare levels in Uganda.

Facility level	Diagnostic capabilities	Microscopy/Culture findings	Clinical interpretation	Required actions
Health Centre II (Parish)	Clinical assessment only	N/A	Based on symptoms: vulval itching, soreness, offensive discharge, dyspareunia	Syndromic management Refer to Health Centre III
Health Centre III (Sub-county)	Clinical assessment + Basic microscopy (Potassium hydroxide (KOH) Wet mount, gram stain)	Blastospores + Pseudohyphae + Neutrophils = Active *Candida* infection (likely *C. albicans*) Blastospores + Neutrophils (No Pseudohyphae) = Possible n*on-albicans Candida* infection *Candida* Elements Present, No Neutrophils = Likely candida colonization, no infection No *Candida*, No Neutrophils = No evidence of *Candida* infection Neutrophils Present, No *Candida* = Infection is present but may not be *Candida* (consider bacterial vaginosis or aerobic vaginitis)	Identify and differentiate acute from recurrent Vulvovaginal candidiasis	Treat acute cases, refer recurrent cases to Health Centre IV
Health Centre IV, Private hospitals, & District Hubs	Microscopy & fungal culture, chromogenic identification to at least *C. albicans* or *non-albicans*, susceptibility testing to fluconazole (Kirby buer disk diffusion method)	Fluconazole susceptible / resistant candida	Species identification, susceptible / resistant to fluconazole	Initiate targeted therapy, refer fluconazole resistant isolates to regional and national labs
Regional/National Labs	Advanced diagnostics (Sugar assimilation biochemical tests, Mass spectrometry, polymerase chain reaction, and sequencing), Minimum inhibitory concentration	Species differentiation & antifungal susceptibility testing	Monitor antifungal resistance trends	Guide national treatment protocols, support lower-level labs

Health Center III facilities operating at the sub-county level would conduct more comprehensive clinical examinations and basic microscopy, including 10% Potassium hydroxide (KOH) wet mount and Gram stain analyses. These facilities would play a crucial role in classifying cases as acute or recurrent, guiding appropriate treatment and referral decisions.

Hub laboratories and higher-level facilities would provide advanced diagnostic capabilities, including culture-based species identification and fluconazole susceptibility testing. These centers would also implement robust quality control measures to ensure reliable and consistent results across the network **(**S3 Image). Regional referral hospitals could further enhance this system by providing molecular testing capabilities, conducting epidemiological surveillance of antifungal susceptibility patterns, and offering technical support to lower-level facilities.

### Quality assurance and sustainability

The success of this implementation framework depends heavily on establishing standardized protocols across all facilities, supported by regular proficiency testing and comprehensive equipment maintenance programs. Continuous training and supervision of healthcare workers, combined with cost-effective resource allocation, would ensure the long-term sustainability of these improved diagnostic services.

### Monitoring and evaluation

Effective implementation requires careful monitoring of key performance indicators, including the correlation between microscopic and culture results, species distribution patterns, and antifungal susceptibility trends. Regular assessment of turnaround times and clinical outcomes would provide valuable feedback for the continuous improvement of the system.

## Conclusion

The implementation of the British Association for Sexual Health and HIV 2019 guidelines through Uganda’s laboratory hub network presents a practical approach to standardizing the diagnosis of VVC. This framework has significant potential to improve diagnostic accuracy and enhance antifungal stewardship, ultimately leading to better patient outcomes. The success of this initiative relies on sustained resource allocation, robust quality control measures, and systematic monitoring of implementation progress

## Declaration of competing interest

The authors have no competing interests to declare.

## Appendix Supplementary materials

Supplementary information S1 file. Forest Plot of VVC Prevalence Among Women in Uganda. Authors (Year): The names of the study authors and the year the study was published; District - The geographical district where the study was conducted; Population - The population sampled in the study; sample size: the total number of participants in the study. VVC Cases - The number of vaginal viral carriage cases observed. pdf Image, application 1

S2 Image. Map of Upgraded Laboratory Hubs in Uganda. This map illustrates the locations of the 100 upgraded laboratory hubs across Uganda, strategically positioned to serve surrounding health facilities within a 40-kilometer radius. These hubs could enhance the diagnostic capacity and provide vital support to lower-level health centers, addressing key challenges in the diagnosis and treatment of conditions such as Vulvovaginal Candidiasis (VVC). tif Image, image 2

S3 Image. A comprehensive adaptation framework that addresses both the technical and operational aspects of implementing guidelines while ensuring sustainability and effectiveness across all levels of care. tif Image, image 3
