# Supplementary material for: Standardizing Vulvovaginal candidiasis diagnosis in Uganda: A case for British Association for Sexual Health and HIV 2019 guidelines
Source: IJID Reg. 2025 Aug 21;16:100735. doi: 10.1016/j.ijregi.2025.100735 (PMC12445571; doi:10.1016/j.ijregi.2025.100735)

Studies reporting Vulvovaginal candidiasis in Uganda

Study Selection

A systematic search identified studies reporting the prevalence of vulvovaginal candidiasis (VVC) in Uganda. Peer-reviewed journal articles and institutional reports published from inception to January 2025 were considered. Eligible studies had to report VVC prevalence among Ugandan women, use microscopy and/or culture-based methods for diagnosis, and clearly define sample size and diagnostic criteria.

Data Extraction and Quality Assessment

Authors (Year)	District	Population	Sample size	VVC Cases	Proportion
Jonani et al (2024)	Kampala	Pregnant women	154	79	0.51298701
Mtende et al (2024)	Hoima	Non pregnant women	288	77	0.26736111
Mugisha et al (2013)	Kampala	Pregnant women	271	199	0.73431734
Mujuzi et al (2023)	Kampala	Women	361	120	0.33240997
Mukasa et al (2015)	Mbarara	Pregnant women	456	207	0.45394737
Richard Mula (2023)	Kamuli	Pregnant women	150	62	0.41333333
Watsemwa et al (2019)	Mbale	Pregnant women	249	126	0.5060241

Statistical Analysis

A random-effects meta-analysis was performed to estimate the pooled prevalence of VVC with 95% confidence intervals (CIs). Heterogeneity was assessed using I^2 statistic. Data analysis was conducted using R (meta package). Forest plots visualized individual and pooled prevalence estimates.

Results Summary

The meta-analysis included 7 studies, with a pooled VVC prevalence of 46% (95% CI: 35–57%). Heterogeneity was substantial ($I^2 = 95.8\%$, $p < 0.0001$), indicating variability in study populations, diagnostic methods, and regional healthcare access.

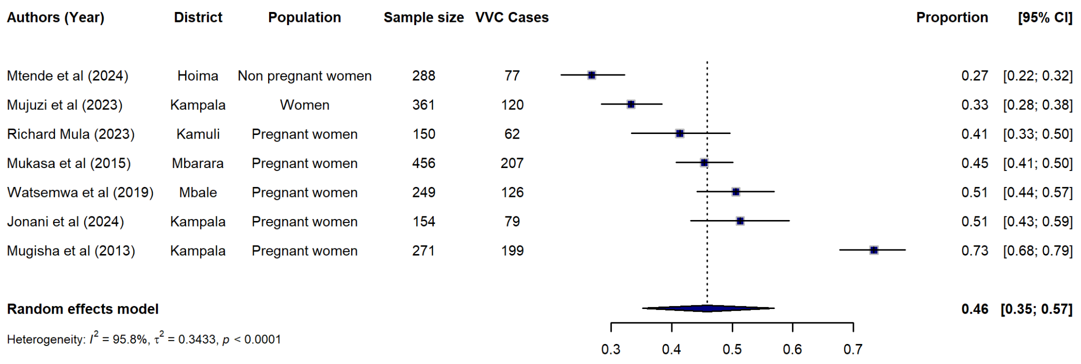

Supplement: Supplementary file 1 — Supplementary information S1 file. Forest Plot of VVC Prevalence Among Women in Uganda. Authors (Year): The names of the study authors and the year the study was published; District - The geographical district where the study was conducted; Population - The population sampled in the study; sample size: the total number of participants in the study. VVC Cases - The number of vaginal viral carriage cases observed. pdf [file mmc1.pdf]
