# Supplementary figures and images for: Standardizing Vulvovaginal candidiasis diagnosis in Uganda: A case for British Association for Sexual Health and HIV 2019 guidelines
Source: IJID Reg. 2025 Aug 21;16:100735. doi: 10.1016/j.ijregi.2025.100735 (PMC12445571; doi:10.1016/j.ijregi.2025.100735)

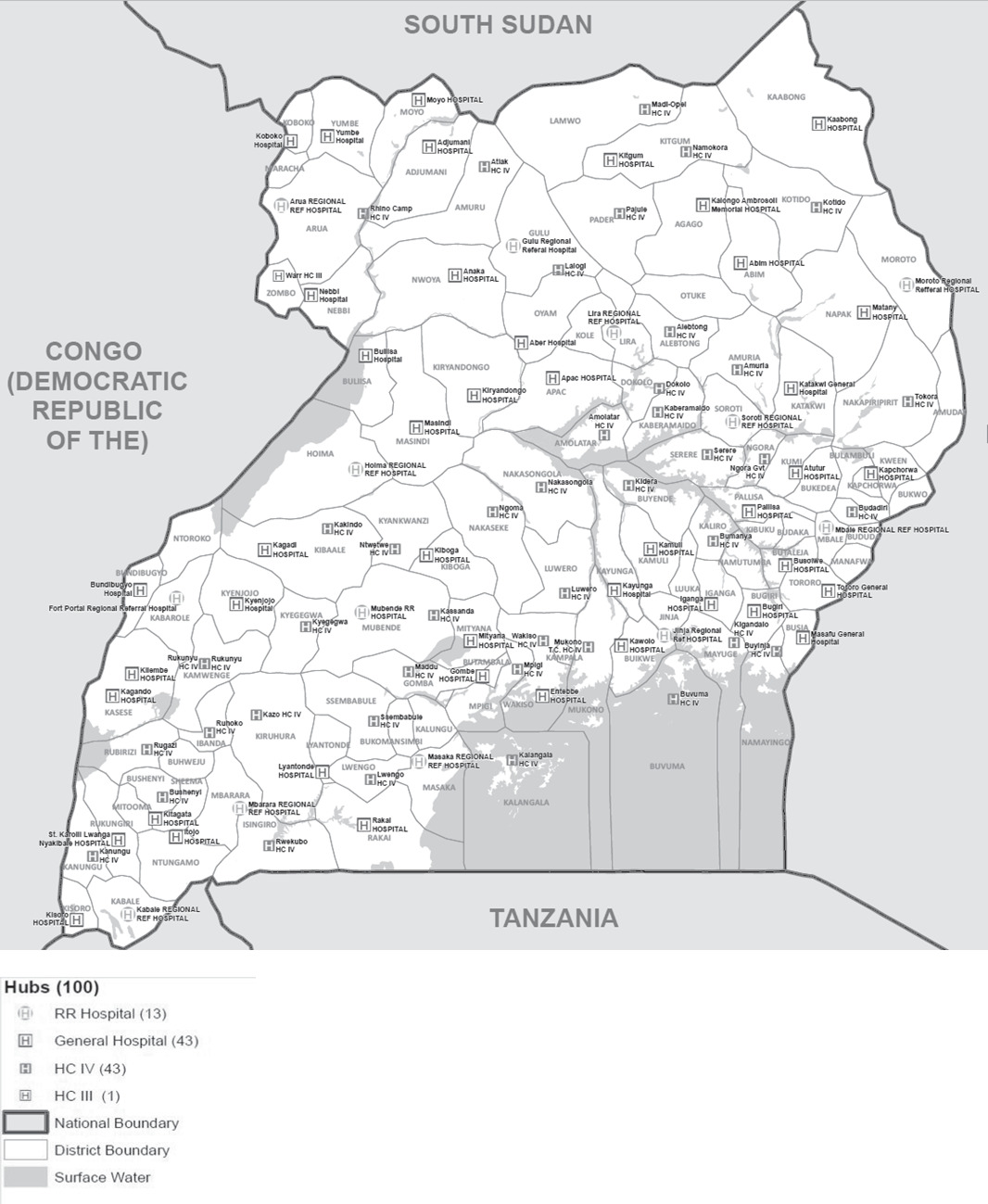

Supplement: Supplementary file 2 — S2 Image. Map of Upgraded Laboratory Hubs in Uganda. This map illustrates the locations of the 100 upgraded laboratory hubs across Uganda, strategically positioned to serve surrounding health facilities within a 40-kilometer radius. These hubs could enhance the diagnostic capacity and provide vital support to lower-level health centers, addressing key challenges in the diagnosis and treatment of conditions such as Vulvovaginal Candidiasis (VVC). tif [file mmc2.jpg]

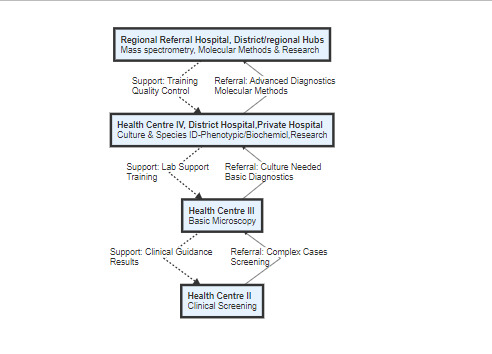

Supplement: Supplementary file 3 — S3 Image. A comprehensive adaptation framework that addresses both the technical and operational aspects of implementing guidelines while ensuring sustainability and effectiveness across all levels of care. tif [file mmc3.jpg]
